# Isolation and detection of *Corynebacterium pseudotuberculosis* in the reproductive organs and associated lymph nodes of non-pregnant does experimentally inoculated through intradermal route in chronic form

**DOI:** 10.14202/vetworld.2015.924-927

**Published:** 2015-07-29

**Authors:** Nur Amirah Abdul Latif, Faez Firdaus Jesse Abdullah, Aishatu Mohammed Othman, Adza Rina, Eric Lim Teik Chung, Mohd Zamri-Saad, Abdul Aziz Saharee, Abdul Wahid Haron, Mohd Azmi Mohd Lila

**Affiliations:** 1 Department of Veterinary Clinical Studies, Faculty of Veterinary Medicine, Universiti Putra Malaysia, 43400 Serdang, Selangor, Malaysia; 2 Research Centre for Ruminant Disease, Universiti Putra Malaysia, 43400 Serdang, Selangor, Malaysia; 3 Department of Veterinary Pathology and Microbiology, Faculty of Veterinary Medicine, Universiti Putra Malaysia, 43400 Serdang, Selangor, Malaysia

**Keywords:** *Corynebacterium pseudotuberculosis*, chronic form, reproductive organs, lymph nodes, detection

## Abstract

**Aim:**

*Corynebacterium pseudotuberculosis* is the etiological agent of caseous lymphadenitis that affects sheep and goats. This study was designed to determine the presence of the causative organism in the female reproductive organs and associated lymph nodes in non-pregnant does experimentally inoculated through intradermal route in the chronic form.

**Materials and Methods:**

18 non-pregnant healthy Katjang does aged 2-year-old were divided randomly into two groups. The first and second group consists of nine non-pregnant does each and the two groups were subdivided into three subgroups. The first group was experimentally inoculated with 1 ml of 10^7^cfu of live *C. pseudotuberculosis* through intradermal route, whereas the second group was inoculated with 1 ml phosphate buffer saline (pH 7) solution intradermally. The first group were further subdivided into three subgroups where, the first subgroup (B1) were kept for 30 days post-infection, second subgroup (B2) were kept for 60 days post-infection, and third subgroup (B3) were kept for 90 days. The second group was further subdivided into three subgroups (C1, C2, and C3) where they were kept for 39, 60, and 90 days post-infection, respectively.

**Results:**

From this study, there was successful isolation of *C. pseudotuberculosis* from the reproductive organs of the treatment group after 60 days post-infection. The subgroups (B1, C1, C2, and C3) did not show any presence of the causative organism in the reproductive organs. The second subgroup B2 and third subgroup B3 showed positive isolation of the causative organisms from the ovary, uterine horns, uterus, cervix, vagina, and inguinal lymph node of the experimental non-pregnant does.

**Conclusion:**

This study showed that chronic infection of *C. pseudotuberculosis* via intradermal route may cause effect toward the reproductive organs and may be able to influence the reproductive efficiency of the infected animals.

## Introduction

Caseous lymphadenitis (CLA) of goats, caused by *Corynebacterium pseudotuberculosis*, has been a significant disease in the majority of goats-rearing regions for over a century. This cheesy gland disease can potentially threaten the Malaysian livestock industry, where the economic loss is significant [1]. This is mainly due to the reduction of wool, meat, and milk production, decrease reproductive efficiencies of affected animals and condemnation of carcasses and skins in abattoirs [2,3].

*C. pseudotuberculosis* organism is a facultative, Gram-positive, and intracellular coccobacillus consisting of two biotypes: A nitrate positive group infecting only horses and nitratenegative group ­infecting only goats and sheep [4]. The *C. pseudotuberculosis* infection occurs after it penetrates into the skin or mucous membranes of susceptible hosts. Most infection of *C. pseudotuberculosis* occurs through a direct contact with purulent exudates that came from rupturing of external and pulmonary abscesses [5]. Skin abrasions due to ear tagging, castration, shearing, docking, and by environmental hazards such as nails, wired fences, splintered wood, and metal edges are the possible indicator for CLA transmission within a herd [6]. *C. pseudotuberculosis* lacks of a tendency to multiply in the environment. However, the bacteria, *C. pseudotuberculosis* can survive for 2-8 months, or more in the environment and any skin abrasions help in persistence transmission of this disease [5].

According to Dercksen *et al*. [7] CLA is an economically important zoonotic disease of small ruminants worldwide and is characterized by the formation of abscess in the peripheral lymph nodes and in the lungs. Dorella *et al*. [5] explained in detailed that the most frequent form of the disease is characterized by the formation of abscess superficially in lymph nodes and in subcutaneous tissues. Dorella *et al*. [5] also added that these abscesses can also develop internally in organs such as in lungs, liver, kidneys, and spleen, characterizing visceral CLA. Taking into an account, due to lack of information on CLA infection in the reproductive organ of goats, their associated lymph node and its relation with the pituitary gland, this study was conducted.

Clinical identification of *C. pseudotuberculosis* in CLA affected goats using serodiagnostic tests are either lack of specificity or sensitivity [8]. Despite that, enzyme-linked-immunosorbent assay (ELISA) seems to be effective in controlling and eradicates CLA disease in affected herds [7]. However, some of ELISA tests are influence by vaccination in animal [9]. Furthermore, it costs a lot of money to run an ELISA tests for more than 96 samples. Hence, ELISA test cannot give a promising results and it is quite expensive to use by the farmers but reasonable to be use in research area [10]. Other than that, other promising diagnostic test would be polymerase chain reaction (PCR) that are widely use in research area and in livestock farm that in need for identification of *C. pseudotuberculosis* in clinical samples [11]. PCR is not involving a lot of steps to get the promising results. Hence, PCR always be the suggested method in detecting CLA in disease animals [5].

Khuder *et al*. [12] stated that *C. pseudotuberculosis* and its exotoxin (phospholipase D) able to cause disruption in reproductive hormones in mice model. There is a paucity of information in isolation of this organism from the reproductive tract of female goats in chronic infection. PCR has become one of the diagnostic tool in isolation and identification of *C. pseudotuberculosis* where this method owing to its advantage of specificity and sensitivity [13]. Therefore, this study was designed to determine the presence of the causative organism in the female reproductive organs and associated lymph nodes in non-pregnant does experimentally challenged with *C. pseudotuberculosis* intradermally for chronic form.

## Materials and Methods

### Ethical approval

The experimental procedure was conducted under the approval of the Animal Care and Use Ethics Committee reference number (UPM/IACUC/AUP-R29/2014)Universiti Putra Malaysia as required in Malaysia by the Animal Welfare Act (2014).

### Animals and management

Eighteen healthy non-pregnant Katjang does aged 2-year-old with an average weight of 30±5kg were used in this study. The non-pregnant does were acclimatized for 2 weeks prior to the experiment and were fed with commercial goat pellets (300g/goats/day) and cut Napier grass. Swab samples from the nasal, vagina and oral mucosa, and blood via jugular venipuncture were collected for screening of *C. pseudotuberculosis* prior to the experiment. The non-pregnant does were randomly divided into two groups (Group 1 and Group 2) of nine non-pregnant does each group. The first group were further subdivided into three subgroups where the first subgroup (B1) were kept for 30 days post-infection, second subgroup (B2) were kept for 60 days post-infection and third subgroup (B3) were kept for 90 days post-infection. The second group was also further subdivided into three subgroups (C1, C2, and C3) where the non-pregnant does were kept for 30, 60 and 90 days post-infection respectively. The first group was experimentally inoculated with 1 ml of 10^7^ cfu/ml live *C. pseudotuberculosis* whereas the second group was inoculated with 1 ml of phosphate buffer saline (PBS) pH (7) via intradermal route at the neck region.

### C.pseudotuberculosis inoculum preparation

*C. pseudotuberculosis* colony that was previously isolated from an outbreak of clinical caseous lymphadenitis cases among goats at Universiti Putra Malaysia was used in this study. The organism were isolated and subcultured onto newly prepared blood agar media and incubated at 37°C for 48 h. Twenty colonies were inoculated into 500mL of brain heart infusion broth and incubated at 37°C for 48h. The Alcamo (1998) Plate count method was used to determine the bacteria concentration where 10^7^cfu/ml was used in this study.

### Does and inoculation

Twelve non-pregnant Katjang does were divided randomly into two equal groups. The group (1) were inoculated with 10^7^ colony forming unit/1 ml of live *C. pseudotuberculosis* through intradermal route at the neck region and the second group (2) were given 1 ml of PBS intradermal and serves as the control. Following infection, the animals were observed daily for clinical signs for 90 days.

### Sampling and culture

For three consecutive months of post-treatment, blood samples were collected periodically and subjected for further analysis. The non-pregnant does were slaughtered at 30, 60, and 90 days post-infection and the reproductive organs (ovary, uterus, uterine horn, cervix, and vagina) and inguinal lymph nodes were collected. Swab samples were taken all these organs and were cultured in blood agar at 37°C for 48 h. Any suspected growth of *C. pseudotuberculosis* was subjected to PCR analyses.

### PCR condition

The PCR was performed in SensQThermocycler machine and was set with 30 cycles of amplification, following an initial denaturating step at 94°C for 5 min. Each cycle involved denaturation at 94°C for 1 min, annealing at 56°C for 1 min, synthesis at 72°C for 2 min, and final synthesis at 72°C for 2 min. For each PCR tube, a total of 50 µl reaction volume containing 2 µl DNAzol as template DNA, 2 µl of 25 Mm MgCl_2_, 2 µl of ×10 Taq buffer with (NH_4_)_2_SO_4_, 0.5 µl of 10 mMdNTP mix, 0.5 µl forward primer, 0.5 µl reverse primer, 0.5 µl Taq DNA polymerase (5 u/µl), and 42 µl sterile distilled water were added respectively. The amplified products were analyzed by electrophoresis on a 1% (w/v) agarose gel with the addition of 1.5 µl FloroSafe DNA stain and run at 60 volts (V), 350 milliAmpere (mA) for 68 min.

### Primer design

The oligonucleotide primer used in this study was 16S rRNA gene [11]. The forward primer used was (5’CCGCACTTTAGTGTGTGTG’3) and the reversed primer was (5’TCTCTACGCCGATCTTGTAT’3) respectively. The PCR products were detected as *C. pseudotuberculosis* according to the molecular size of 816 bp as documented [11].

## Results

Group 2 (C1, C2, and C3) and Group 1 (B1) showed negative detection of *C. pseudotuberculosis* from all reproductive organs (ovary, uterus, uterine horn, cervix, and vagina) and inguinal lymph node after 30 days post-infection. All non-pregnant does from group B3 showed positive results of detection *C. pseudotuberculosis* from all reproductive organs and inguinal lymph node after 90 days post-infection. For Group B2 only two out of three non-pregnant does showed positive detection of *C. pseutoberculosis* from reproductive organs whereas for inguinal lymph node all three non-pregnant does were positive with the causative organism after 60 days post-infection. The results are summarized in Tables-1 and 2.

**Table-1:** Detection of *Corynebacterium pseudotuberculosis* in Group 1 of non-pregnant Katjang does inoculated intradermally.

Organs	Subgroup	B1			B2			B3		
			
	Goat	G1	G2	G3	G4	G5	G6	G7	G8	G9
Ovary	-	-	-	-	+	+	-	+	+	+
Uterine horns	-	-	-	-	+	-	-	+	+	+
Uterus	-	-	-	-	+	-	-	+	+	+
Cervix	-	-	-	-	+	-	-	+	+	+
Vagina	-	-	-	-	+	-	-	+	+	+
Inguinal lymph node	-	-	-	-	+	+	+	+	+	+

**Table-2:** Detection of *Corynebacteriumpseudotuberculosis* in Group 02 of non-pregnant Katjang does inoculated intradermally.

Organs	Subgroup	C1			C2			C3		
			
	Goat	G10	G11	G12	G13	G14	G15	G16	G17	G18
Ovary	-	-	-	-	-	-	-	-	-	-
Uterine horns	-	-	-	-	-	-	-	-	-	-
Uterus	-	-	-	-	-	-	-	-	-	-
Cervix	-	-	-	-	-	-	-	-	-	-
Vagina	-	-	-	-	-	-	-	-	-	-
Inguinal lymph node	-	-	-	-	-	-	-	-	-	-

## Discussion

*C. pseudotuberculosis* is the causative agent of CLA in ruminants [12,13]. Although the bacterium causes low mortality, but it may influences the fertility of the infected animal, which may lead to major economic losses [14]. Infertility is one of the major problem dealt by goat industry where anything interferences with the reproductive system will lead to an infertile animal [15]. Bacterial infection is one of the causes for reproductive failure in ruminants [16]. Othman *et al*. [17] have reported that does infected with *Corynebacterium pseudotuberculosis* for a period of 30 days able to cause disruption in the reproductive hormones where the change of the hormones resembles pseudopregnancy in a doe. To date, there is a paucity of information related to chronic infection of *C. pseudotuberculosis* in does related to reproductive organs. The results of this study were added knowledge to this field. From this study all the treatment groups showed presence of causative organism in the inguinal lymph nodes after 30 days post-infection until 90 days post-infection and this result is in agreement with Dorella *et al*. [5] who stated the bacteria disseminates freely in the lymphatic system. The presence of this organism in the reproductive organs of the infected does after 60 and 90 days post-infection may cause infertility where according to Jesse *et al*. (2014), stated that presence of the bacterium in the reproductive organs may alter the secretion of female reproductive hormone [13,14].

The result of this study for 30 days post-infection is not in agreement with Othman *et al*. [17] stated that the researcher believed presence of the causative organism in the reproductive organs may cause the changes in the reproductive hormone profiles in their study. The present study, report for the 1^st^ time, the presence of the causative agent in the reproductive organs and inguinal lymph nodes after 60 and 90 days post-infection with *C. pseudotuberculosis* via the intradermal route. The findings from this study differ from a study conducted by Valli and Perry [18] stated reproductive organs are the least frequently affected organs during CLA infection. Hence, this study indicates possible transmission of *C. pseudotuberculosis* in reproductive organs and associated lymph nodes during prolong infection that may cause a reproductive failure that can contribute to the economic loss.

## Conclusion

In conclusion, this study revealed the presence of the causative organism in the reproductive organs and associated lymph nodes of non-pregnant does inoculated via intradermally in the chronic form. These findings may explain the infertility among does infected with CLA for a long period of time.

## Authors’ Contributions

FFJA, MZS, AAS, AWH, and MAML conceptualized and supervised the research. NAAL, AO, AR, and ELTC collected samples, drafted the manuscript and ran all statistical tests. All authors have read and approved the final manuscript.
